# Key Informants’ Perspectives on Childhood Obesity in Vietnam: A Qualitative Study

**DOI:** 10.1007/s10995-022-03502-6

**Published:** 2022-07-26

**Authors:** Tuyen Nguyen, Tiffany Trat, Ngoc Thanh Tieu, Linda Vu, Karen Sokal-Gutierrez

**Affiliations:** 1 University of California San Francisco (UCSF) School of Medicine, University of California (UC) Berkeley-UCSF Joint Medical Program, San Francisco, USA; 2 University of California San Francisco (UCSF) School of Dentistry, Global Children’s Oral Health and Nutrition Program, University of California Berkeley School of Public Health, San Francisco, USA; 3 Global Children’s Oral Health and Nutrition Program, University of California Berkeley School of Public Health, Berkeley, USA; 4 University of California (UC) Berkeley-UCSF Joint Medical Program, University of California Berkeley School of Public Health, Berkeley, USA

**Keywords:** Child obesity, Diet, Physical activity, Malnutrition, Vietnam

## Abstract

**Objectives:**

Vietnam’s post-war globalization, economic development, and urbanization have contributed to a nutrition transition from traditional diets to highly-processed diets, and increased prevalence of childhood overweight and obesity. Our study aims to explore the attitudes and behaviors driving this epidemic.

**Methods:**

This qualitative study focused on the perspectives and practices of Vietnamese parents, schoolteachers and doctors. Semi-structured interviews were conducted with a convenience sample of 12 regarding the historical, social, and cultural influences contributing to childhood overweight and obesity. Audio-recorded interviews were translated and transcribed, then analyzed using modified ground theory to identify themes and representative quotes.

**Results:**

Five themes were identified: (1) Change in diet between generations, (2) Preference for rounder children, (3) Unhealthy feeding practices, (4) Reduced physical activity, and (5) Increasing awareness of childhood obesity. A conceptual map detailed the shift from war-time to post-war economic environment and psycho-social context for raising children to be large, safe and academically-successful.

**Conclusions for Practice:**

We found that globalization, urbanization and economic development—in the context of historical, social and cultural attitudes—may contribute to increasing child obesity in Vietnam. Obesity prevention through public health and educational institutions should involve policies and programs for healthy eating and exercise, tailored to address parental figures’ concerns.

## Significance Statement

Rates of child obesity in Vietnam have increased dramatically over the past two decades. Previous existing qualitative assessments for this increased prevalence prevail, and our findings show how Vietnam’s post-war economic development, globalization and urbanization—in the context of deep-seated historical, social and cultural attitudes—may contribute to risk for child obesity and other non-communicable diseases. We identified the need to tailor policies and interventions to promote healthy diet and physical activity to support parents’ and grandparents’ fears and concerns.

## Introduction

For many low- and middle-income countries (LMIC), childhood obesity is an emerging problem. Many countries exhibit the “double-burden of malnutrition”, in which childhood underweight and overweight both exist (Poskitt, 2009; Schott et al., 2019). Though nutrition research in LMICs historically focused on malnutrition in terms of undernutrition, the World Health Organization’s 2016 policy report addresses overweight and obesity as a growing global health challenge with urgency for the United Nations to act promptly. In the same report, the World Health Organization adapts the definition of malnutrition to include “undernutrition (wasting, stunting, underweight), inadequate vitamins or minerals, overweight, obesity, and resulting diet-related non-communicable diseases” (WHO, 2017). Childhood obesity is linked to both physical and psychological consequences including cardiovascular disease (Bridger, 2009), type 2 diabetes (Hannon et al., 2005), and depression (Rankin et al., 2016).

Because of its rapid growth and economic boom over the last few decades, Vietnam is experiencing an accelerated nutrition transition (Khan & Khoi, 2008) leading to an increase in availability of processed snack foods, fast foods, and sugar-sweetened beverages (Vo & Francic, 2017). In fact, it is the 13^th^ largest export market for food and beverages from the United States (Vo & Francic, 2017). In Vietnam, these dietary and behavioral changes are most prominent in urbanized areas (Trang et al., 2012).

With the increasing recognition of childhood obesity as an emerging public health issue in Vietnam, there has been a surge in studies on childhood overweight and obesity over the last ten years. In Ho Chi Minh City, a cross-sectional study of 10,949 children in 2012–2014 found the prevalence of overweight and obesity in primary school children to be 20–30%, more than double the prevalence found ten years earlier in 2002–2004 (Mai et al., 2020). Similarly, a study of pre-school children in Hanoi found the prevalence of overweight and obesity to be 16.7% and 4.5%, respectively (Do et al., 2017). Rates of childhood obesity in Vietnam have been found to be higher amongst boys from wealthier families living in urban areas (Pham et al., 2020). Additionally, risk factors associated with higher rates of obesity were diets with high-calorie foods and reduced physical activity (Do et al., 2015a, 2015b). Vietnam is also experiencing a rise in nutrition-related chronic diseases. A systematic review by Nguyen et al. shows an alarming increase in type 2 diabetes in Vietnam with risk factors including higher levels of body and abdominal fat, and sedentary lifestyle (2015).

While epidemiological trends are well-documented, there is a lack of qualitative data to understand the attitudes and behaviors driving the increase in childhood obesity. The purpose of this study is to gain a better understanding of childhood overweight and obesity in the context of historical, social, and cultural influences from the perspectives of key informants including community leaders, health care providers, and teachers. The data from this study will be useful to inform development of intervention programs and public health campaigns to reduce childhood overweight and obesity in Vietnam.

## Methods

This is a qualitative study of interviews with a convenience sample of key informants including community leaders, health care providers, and teachers in Vietnam to explore the historical, social and cultural contributors to child obesity in urban Vietnam. We collaborated with Vietnam-based organizations—East Meets West Foundation in Da Nang and University of Medicine and Pharmacy in Ho Chi Minh City. Ethical review and approval in accordance with the Declaration of Helsinki and its amendments were obtained from the University of California Berkeley Committee for Protection of Human Subjects (#2011-04-3176) and University of California San Francisco (#585-1) by the UC Reliance System. We have followed the COREQ criteria for reporting qualitative research throughout this research study (Tong et al., 2007). The Vietnam partner organizations reviewed the protocol and agreed to rely on the US Institutional Review Board approval, and signed a Memorandum of Understanding.

Data collection took place during the summer of 2016 in Ho Chi Minh City and Da Nang. Twelve participants were recruited by our partner organizations, with 8 from Da Nang and 4 from Ho Chi Minh City. Participants included children’s health non-profit organization project managers, a pediatrician, a dentist, a public health university professor, and pre-school teachers. Participants were ages 30–47 and were all parents themselves.

Participants provided both verbal and signed informed consents, and completed a short demographic survey before the interview. Participants were provided informed consent forms translated into Vietnamese. A semi-structured interview guide addressed four domains identified from literature review on child obesity in LMICs: nutrition transition, children’s diet, parental feeding practices, and child physical activity and health. The semi-structured guide facilitated a systematic approach to questions with further exploration of topics that arose during the interview (Harvey-Jordan & Long, 2001). To help reduce bias, one interviewer conducted all 12 interviews. Interviews were 20–40 min long, conducted in Vietnamese, and audio-recorded.

The recordings were securely de-identified, transcribed verbatim, and translated by three co-authors. These co-authors are advanced English and Vietnamese-native speakers and are also proficient in reading and writing in these languages. A modified grounded theory approach was used to analyze the transcripts. Grounded theory is a systematic methodology used in social sciences and public health to categorize concepts and develop explanatory theories from qualitative data (Corbin & Strauss, 1990). In our modified approach, we first reviewed a small number of transcripts to identify and code concepts. All co-authors reviewed separately, then discussed as a group to refine codes and create a codebook using “axial coding” to evaluate the relationships among the codes to organize into themes and subthemes by inductive and deductive reasoning (Corbin & Strauss, 1990; Vollstedt & Rezat, 2019). Co-authors then independently evaluated transcripts using the codebook, and tagged new themes or sub-themes that emerged. Additional reflective practices including consulting with colleagues and mentors were used throughout the analysis phase (Cutcliffe, 2003). Supporting quotations were chosen from the interviews to illustrate the themes and subthemes. Three co-authors quantified the occurrence of themes and subthemes across all transcripts.

## Results

Five themes were identified regarding the historical, social and cultural contributors to childhood obesity in Vietnam. Table 1 highlights the themes, subthemes, and average counts from coding the themes that were identified.

**Table 1:** Themes and subthemes

Themes	Subthemes	Average counts
**Theme 1** Change in diet between generations	Economic growth, globalization, and urbanization post Vietnam war	44
	Change from large families and food scarcity to smaller families and food abundance	
	Change from traditional rural diet to urbanized processed foods and fast food diet	
**Theme 2** Preference for “rounder” children	Parents define “skinny” as “unhealthy”	36
	Parents believe boys need more food and should be “taller” and “larger”	
	Using endearing terms to describe overweight/obese children	
	Associating “rounder” appearance with higher socioeconomic status and ability to support their family	
**Theme 3** Unhealthy feeding practices	Increased frequency and overfeeding during meals and snacks	100
	Promotion of sugar-sweetened milk as healthy	
	Increased access to sugar-sweetened snacks & beverages and fast food	
**Theme 4** Reduced physical activity	Parents perceive urban environment and traffic to be unsafe for children to walk to school or play outdoors	49
	Children spend free time studying instead of playing due to increased academic pressure	
	Increased sedentary behavior in children due to use of electronics/new technologies	
**Theme 5** Increasing awareness of child obesity	Parental action to limit sweets and overfeeding to prevent overweight/obesity	43
	Increased parental concern about accessibility of sugar-sweetened foods and beverages at school	
	Parental hopes for increased government action to prevent overweight/obesity	

### Theme 1: Change in Diet Between Generations

Participants described a generational shift in children’s diet, particularly post-war Vietnam. This change was primarily attributed to changes in the economic and food environment, and the resulting fears and desires of parents regarding their children’s diet and nutrition status. Parents described their own wartime and immediate post-war upbringing dominated by economic and food scarcity, large families, fear of child malnutrition, small portions, farmed foods, and drinking water. One parent recollected:

We didn’t have much money. My house had ten kids. We would wait until morning to see what there was to eat, and we would eat that… We ate because we had food and needed to eat, not because we were trying to get full.

In contrast, parents described how children now are being raised in an environment of economic growth—especially in cities—with greater income for working parents, greater availability of natural and processed foods and drinks, smaller families, and intense focus from parents and grandparents to ensure that children eat and grow. One doctor explained:

A long time ago, we had less choices in the foods that we ate. Now there are a lot more types of foods to eat and drink.

A teacher added:

A family won’t give birth to many kids... However, when you have only one child, your worries for them turn into fears—are they ugly, in pain, skinny? One kid with two parents, two grandparents on the maternal side, and two grandparents on the paternal side, then there are six people working to give that child whatever they need to be as healthy as possible.

### Theme 2: Preference for Rounder Children

Based on their upbringing, parents believed that children who were skinny were unhealthy, and they feared malnutrition in their own children. Parents described feeding their children with high-protein food until they felt full, especially boys. One mother discussed the difficulties of feeding her son:

I was scared he wouldn’t have enough to eat and would be malnourished... ‘You have to eat! Eat!’ Not only did I have a hard time, my son also had a hard time eating. I told him he can’t be skinny.

Parents described their preference for having “rounder” children who would be viewed as “healthy.” Endearing Vietnamese terms such as *dễ thương* (meaning “easy to love”or “cute”) and *mập* (meaning “fat" or “chubby”) were used to justify this preference. Parents refrained from using the formal word for overweight/obese, *béo phì*, to refer to their children since the term is considered offensive. A health professional described how larger children reflect a higher socio-economic status and good parenting:

There’s an inherent flaw in our culture. It’s an old way of thinking. We think round children are ideal. It’s a reflection of their wealth, their ability to support. The only way a child can be round is if you are rich.

### Theme 3: Unhealthy Feeding Practices

Economic prosperity, urbanization, food abundance, and a preference for rounder children, contributed to the trend of overfeeding children. On a daily basis, key informants reported that children ate more than 4 meals, drank sweetened milk up to 5–6 times, and consumed unhealthy snacks and beverages. On special occasions, families celebrated with fast food meals. One doctor summarized:

Kids start with eating and drinking sweet foods, then they grow up to like sweet foods.

A teacher highlighted:

…For folks here, when it is their children’s birthday, they are treated to fast food. They go all out.

Particularly in urban areas, the widespread availability of enticing and low-cost processed snacks, sugary drinks, and fast foods made them a daily part of children’s diet at home and outside. One teacher commented:

If they live in the city, it takes only about a minute to reach these foods/drinks… If they live in a story complex, it takes a minute to go down the elevator and buy them. If they live in a [house], they just need to go... next door. If they’re outside, it takes about one to five minutes to walk to the nearest place that sells these foods/drinks.

### Theme 4: Reduced Physical Activity

Many parents discussed physical environments leading to less opportunity for physical activity for children, both at-home and at-school. Whereas parents recalled walking or biking to school and playing outdoors, they are now afraid of their children growing up in an urbanized environment with dangers of traffic and child abduction. With motorcycles or cars, parents feel safer driving their children to school. One teacher recounted:

Back then... Our parents did not tend to us—we walked to school by ourselves… but now the villages and the cities are so crowded. There’s a common fear of kidnapping, so parents are stricter now. We take them to school. If we don’t have a car, we’ll take them on a scooter. There are few people who walk their kids to school.

Parents also described the increased focus on ensuring their children’s academic success. As a result, more time at school and at home is devoted to studying, rather than to sports and physical activity. One doctor described:

There’s little push to exercise, to play sports. There’s a big push to study, to go to school. The kids don’t have time to play sports.

In addition to studying at home after school and on weekends, parents described allowing their children to have substantial screen time for entertainment. One teacher explained:

When they don’t want to be active, they won’t. They often just like to sit, lay around, and be motionless. [My] oldest [child] likes to do this and watch TV.

### Theme 5: Increasing Awareness of Childhood Obesity

Among health and educational professionals, and parents with higher levels of education is an increasing awareness of the adverse consequences of child obesity on children’s health and development. One teacher noted:

On average, a lot of children by twelve months can walk. Now, they struggle with standing up because of how overweight they are. These children generally can only get up when they’re 16 to 17 months old.

Many noted the need for the childhood obesity epidemic in Vietnam to be addressed on a parental-level, school-level, and government-level. One teacher described their efforts:

I don’t let [my child] drink many sugary drinks, but he does like to drink them. [He] will always ask for them, but [my husband and I] just don’t generally let him drink them. [We] prevent him from [having] sugary drinks. We let him drink water only.

Although many parents described their attempts to limit sugar-sweetened foods and beverages at home, they noticed that their efforts were undermined by children’s increased access to these foods and beverages at and around school.

[Schools are] more likely to have foods that the children like to eat, whereas at home, there may be foods not to the children’s liking.

Many parents also recognized that their children needed more physical activity at home and at school to further promote a healthy lifestyle. One teacher said:

There should be more exercise in school, but also in the home. I think this is an issue of great prevalence in Vietnam… This is especially true in wealthy families within the cities.

Parents recognized the role of the government in keeping their children healthy; with this increased access to and abundance of sugar-sweetened foods, one teacher explained where the government could help:

The government just issued a warning about the nutritional values to the public, but there is no policy or law being implemented that provides direction for people to follow.

### Summary of Drivers of Childhood Obesity in Vietnam

**Fig. 1:**
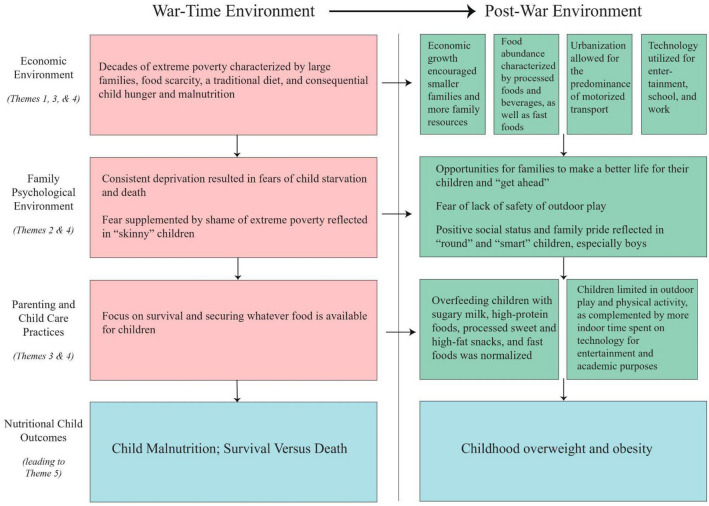
Summarizes the historical, social and cultural context, with the identified themes, that may contribute to childhood obesity in Vietnam. It details how parents’ and caregivers’ war-time experience of extreme poverty, food insecurity, fear of child malnutrition and focus on child survival laid the foundation in the current environment of economic growth, food abundance, urbanization and technology for a drive to overfeed children, keep them safe, and encourage their academic development more than physical activity, contributing to child obesity

Figure 1 summarizes the historical, social and cultural context, with the identified themes, that may contribute to childhood obesity in Vietnam. It details how parents’ and caregivers’ war-time experience of extreme poverty, food insecurity, fear of child malnutrition and focus on child survival laid the foundation in the current environment of economic growth, food abundance, urbanization and technology for a drive to overfeed children, keep them safe, and encourage their academic development more than physical activity, contributing to child obesity.

## Discussion

This qualitative study of a convenience sample of Vietnamese parents, schoolteachers and doctors found five themes associated with child obesity—change in diet between generations, preference for rounder children**,** unhealthy feeding practices**,** reduced physical activity**,** and increasing awareness of childhood obesity. Study participants described the roots for parents’ attitudes and practices in the historical, economic, psychosocial, and cultural influences of their war-time and post-war environments.

Our study supports the findings of other global quantitative and qualitative studies identifying the contributors to child obesity—excessive feeding and insufficient physical activity—with factors at the child, family and community levels (Boonpleng et al., 2013; Galvez et al., 2010). Our findings also support other studies from Vietnam on the local dietary and physical activity risk factors for child obesity (Ngan et al., 2018; Pham et al, 2019, 2020; Trang et al., 2012). In particular, our study supports research from Vietnam on how parents’ and caregivers’ control of feeding practices—both encouraging children to eat more, and also restricting certain foods—may contribute to child obesity (Babington & Patel, 2008; Do et al., 2015a, 2015b, 2016). Our findings also support other studies from Vietnam showing that reduction in physical activity for children is related to parents limiting children’s opportunity to walk to school and play outdoors to help ensure their safety, and commensurately increasing their at-home screen time for learning and entertainment (Leather, et al., 2011; Nguyen et al., 2016).

This study adds to the literature by exploring why Vietnamese parents and caregivers may follow these feeding and physical activity patterns that contribute to child obesity. The parents interviewed in this study were very insightful and open about their observations of Vietnamese history and culture, and their thoughts and feelings about feeding their own children. Their reflections highlighted the background of Vietnamese historical trauma from decades of war and foreign occupation from the 1940s through the 1970s, in which parents and grandparents experienced severe food insecurity and child death from malnutrition (Wertheim-Heck & Raneri, 2020). They recognized that the current cultural value for “round” children was driven by deep-seated fears of their children being too skinny and undernourished; and their motivation to overfeed their children was to bolster their children’s health and for their community to view them as good parents who could provide adequately for their children. A study from England on impulsivity suggests that this response is universal to deprivation (Tunney, 2022). Those who experience early food scarcity, relative deprivation, and economic uncertainty opt in favor of choice parameters that reward them “smaller sooner” rather than “later larger” (Heitmann et al., 2012; Tunney, 2022; Wells, 2006). In context with our study, we find that the parental generation’s historical experience with socioeconomic and food insecurity explain the over-consumptive feeding habits toward their children in present time Vietnam—a period of resource abundance—because they anticipate a possible future period of resource scarcity. While this motive to overfeed their children was driven by a desire to prevent their children’s undernutrition, children consequently may also be at risk for overnutrition and obesity—as expressed by one interviewee considering a desire for larger children as an “inherent flaw.” Well-intentioned parents may aim to protect their children from one health risk (undernutrition), but inadvertently expose them to the opposite health risk (obesity). Additionally, with urbanization and globalization, parents recognized that encouraging their children’s academic success—and spending free time studying instead of engaging in play or sports activities—could help prepare their child for future career success and economic opportunity. Interviewees mentioned both the upside and downside of these overfeeding habits. While overfeeding their children is a protective precaution for underweight growth statuses and taking pride in their children and grandchildren as a representation of how far their lineage has come—wealthwise, in this case—we also witness overnutrition (overweight and obesity), stress from academic performances, and other chronic diseases from the pressure placed on children to eat more. Some of the participating parents did recognize that overfeeding and limiting physical activity were contributing to childhood obesity and poor overall health, and they cited their family’s efforts to encourage healthier eating and physical activity, as well as some government’s efforts to promote better nutrition.

Specifically in Vietnam and other LMIC’s, the nutrition transition and increasing rates of childhood obesity initially appear in higher-income urban families. However, continued economic growth is likely to lead to increasing rates of childhood obesity in middle- and lower-income populations, including in rural areas (Goryakin et al., 2015). A recent study predicted that overweight prevalence among lower-income populations in Vietnam will increase by 80–100% from 2016 to 2040, which will likely contribute to large burdens of other non-communicable diseases, and further stress the public health system (Templin et al., 2019).

This study had some limitations, particularly the convenience sample of key informants drawn only from urban Ho Chi Minh City (South Vietnam) and Da Nang (Central Vietnam), which may reduce the generalizability to families from northern cities, rural areas, lower socioeconomic strata, and ethnic minority populations. Therefore, our participants do not represent backgrounds of all socioeconomic strata and all regions of Vietnam, where our themes may vary or differ among different subgroups in Vietnam. Although the sample size of twelve appears small, the semi-structured interview format enabled deeper exploration of participants’ thoughts and feelings; and the use of multiple translators, transcribers and coders facilitated extensive discussion and analysis of themes. One study asserts that twelve interviews is sufficient to saturate themes—when no new subthemes or themes are observed in the data—and thus, our sample size could be considered appropriate (Guest et al., 2006). Further research diversifying the perspectives of parents and child health and development professionals from other regions, socioeconomic statuses, and ethnicities would be important.

### Implications for Future Interventions and Research

In all, this study supports and adds to previous findings that early childhood parenting practices for over-feeding and limiting physical activity are contributors to childhood obesity, and are influenced by historical, economic, psychosocial and cultural context. Coupled with the rising prevalence of childhood obesity in Vietnam is an urgent need for policies and programs to prevent child obesity, addressing these biases. Policy interventions should ensure that children and families have healthy food environments (e.g., limiting the sugar content of child milk products, limiting the advertising of unhealthy snacks and beverages to children and families, and prohibiting the sale of unhealthy products in and around schools), and safe physical activity environments (e.g., walking, biking, sports and play areas). In addition, public education programs should reach out to families through maternal-child health services, preschools/schools and media with clear messages on child nutrition and physical activity for obesity prevention— with carefully-designed messages to support parents’ and caregivers’ motivations for healthier child-rearing practices—including encouraging traditional diets, avoiding overfeeding, limiting screen time, and encouraging active play.

## Conclusions

Our qualitative study of a convenience sample of Vietnamese parents and child health and development professionals found that globalization, urbanization and economic development—in the context of deep-seated historical, social and cultural attitudes—may contribute to increasing child obesity. To effectively reduce child obesity, public health and educational institutions should advance their efforts to promote policies and programs for healthy eating and physical activity, tailored to address and support parents’ and grandparents’ fears and concerns.

## Data Availability

Not applicable.

## Abbreviations

LMIC: Low and middle income countries
